# Utilizing associational resistance for biocontrol: impacted by temperature, supported by indirect defence

**DOI:** 10.1186/s12898-015-0048-6

**Published:** 2015-05-29

**Authors:** Sari J Himanen, Thuy Nga T Bui, Mengistu M Maja, Jarmo K Holopainen

**Affiliations:** Natural Resources Institute Finland (Luke), Management and Production of Renewable Resources, Production Systems, Lönnrotinkatu 5, FI-50100 Mikkeli, Finland; Department of Environmental Science, University of Eastern Finland, Kuopio Campus, P.O. Box 1627, FI-70211 Kuopio, Finland

**Keywords:** Associational resistance, Herbivory, Host location, Indirect defence, Parasitoids, Semivolatiles, Temperature

## Abstract

**Background:**

Associational herbivore resistance is potentiated by neighbouring heterogenic plant species that impact a focal plant’s attraction to herbivores or the damage that they cause. One mechanism to confer associational resistance is believed to be exposure to neighbour-emitted volatiles, the receivers of which range from intra- and interspecific neighbour plants to higher-trophic-level insects. In previous studies the passive adsorption of neighbour-emitted semivolatiles has been reported, but little is known regarding the mechanisms and ecological consequences on the receiver plant and its associated biota. To utilize volatile-based associational resistance for agricultural applications, it is imperative to know its effectiveness under varying diurnal temperatures and whether herbivore natural enemies, providing biological control, are impacted. Mimicking varying diurnal temperatures in a laboratory set-up, we assessed how the tritrophic model system *Brassica oleracea* var. *italica* (broccoli)–*Plutella xylostella* (crucifer specialist herbivore)–*Cotesia vestalis* (endoparasitoid of *P. xylostella*) is influenced by exposure to the natural semivolatile emitter plant *Rhododendron tomentosum* Harmaja.

**Results:**

*Rhododendron tomentosum-*exposed *B. oleracea* was less susceptible to *P. xylostella* oviposition at both night-time (12°C) and day-time (22°C) temperatures and less favoured and damaged by *P. xylostella* larvae at 12°C. Exposure did not interfere with indirect defence, i.e. attraction of the natural enemy *C. vestalis* on host-damaged, *R. tomentosum*-exposed *B. oleracea* under 22°C, while there was a reduction in attraction (marginal preference towards host-damaged *B. oleracea*) under 12°C.

**Conclusions:**

The ability of *R. tomentosum* exposure to render associational resistance against an agriculturally important *Brassica* herbivore *P. xylostella* without severely compromising the specialist parasitoid *C. vestalis* host location encourages further studies on the potential of using this naturally abundant plant for biocontrol. The generality of our finding on temperature as a potential regulating mechanism for the efficacy of semivolatile emitter-based associational resistance towards specialist pest larval damage should be further studied in natural and agricultural associations. Our study emphasizes the need to develop techniques to compare volatiles at the leaf versus air interface and associate their appearance and ecological role with times of activity and level of specialisation of herbivores and their natural enemies.

## Background

Associational resistance, the protection of a focal plant from herbivory via neighbouring a heterogenic species [1, 2], is an example of how plant–plant interactions might affect plant defence against biotic attackers. Such neighbour-mediated resistance is believed to operate via multiple strategies: the “resource concentration hypothesis” is based on lower host plant density and reduced accessibility to host plants by attacking herbivores in mixed communities [3], and the “enemies hypothesis” predicts that natural enemy abundance is increased when food sources and shelter are provided by neighbours, thereby reducing herbivory [4]. The “semiochemical diversity hypothesis” relies on volatiles disturbing herbivore olfaction, which is used in host location and reproduction [1]. Airborne plant volatiles are important infochemical cues for associated specialist species biota in particular [5]. The host location by insects [6] and the activation of a plant’s inducible defences to attract natural enemies of herbivore attackers [7] to provide indirect defence are partly regulated by volatile signals. The complexity of volatile signalling in multitrophic systems is illustrated by numerous recent findings suggesting novel mechanisms for volatile-mediated interactions. Such examples include the passive adsorption and re-release of neighbours’ defensive volatiles with semivolatile characteristics, i.e. low vaporization rate to the gaseous phase, on the surface of interspecific foliage [8].

Associational resistance as mediated by plant volatiles has recently been demonstrated for a number of associations [8–11]. Associational resistance in general has been utilised for decades in agriculture in the form of mixed cropping with the aim of reducing pest damage [12, 13]. Only in approximately 50% of cases, has pest protection by neighbours been effective in mixed cultures [14]. Specific aromatic plant volatiles have often been tested with success for the purpose of repelling or masking host cues (i.e., olfactory camouflage) towards herbivores in various types of agroecosystems (e.g., [15, 16]), although the active role of the chemical component has also been doubted, and opinions have been raised in favour of companion crops acting as pure physical barriers [17]. Variation in the environmental conditions and specificity by the studied plant-herbivore system can complicate revealing any general underlying mechanisms providing associational resistance alone or in concert with other simultaneously acting interactions [9]. Much remains to be revealed regarding multitrophic interactions that are mediated by volatiles, particularly on their environmental persistence [18–21], potential trade-offs between the many ecological effects to which they contribute [22], and their co-evolution and adaptive value [23]. The utilization of associational resistance for biocontrol in the form of trap crops [24, 25], repellent or host odour-masking companion plants [16], intercropping [15], or synthetic compounds released from dispensers [26] might also benefit greatly from an improved understanding of the mechanisms regulating volatile-mediated associational resistance.

Understanding the concurrent roles and interplay between different volatile-mediated interactions (with the same compound changes potentially impacting multiple interactions simultaneously) is essential when aiming to utilize volatiles in pest protection. For example, a trade-off might exist if the adsorbed compounds, which are emitted by the neighbour and confer associational resistance, interfere with the attraction of natural enemies to suppress herbivore pressure [22]. Thus, when designing biocontrol applications based on volatiles, impacts on both the herbivores [16] and their natural enemies [27–29] and, optimally, the persistence of impacts under abiotic variation as encountered in field environments should be considered. Neighbour volatiles might variably impact natural enemies, as recently shown for ladybirds on potato exposed to onion volatiles: TMTT was an attractant, whereas (*E*)-nerolidol acted as a repellent [30]. If the neighbour-emitted compounds are semivolatile in nature, the impact might be even stronger or more prolonged.

The stability and functional distance of associational resistance is another puzzling issue also when considering airborne plant volatiles in field conditions [21, 31]. Because insect olfaction is believed to be remarkably sensitive [6], small changes in volatile signals may reveal or disguise a great deal of information, making volatile-based defences vulnerable to interference [19]. Associational resistance occurs in nature across a range of abiotic variation, including diurnally and seasonally varying temperature and high precipitation as well as drought episodes and changes in atmospheric constituents, such as carbon dioxide. Of these, the prevailing temperature is well known for its role in the control of diffusion and volatility of volatile compounds and their longevity in the atmosphere [32, 33]. The major C_5_ volatile isoprene is synthesized and emitted in greater amounts under increasing temperatures [34]. For other plant volatiles, such as monoterpenes and sesquiterpenes, the temperature dependence of emission is less obvious. Both physiological and physicochemical regulation takes place in plant foliage and at the leaf-atmosphere interface [35]. In addition, the ability to store these compounds or their precursors in specialized leaf structures (e.g. secretory cavities or trichomes) and their inducibility upon abiotic and biotic stresses creates variance from direct responsiveness to increasing temperature or modelling their species-specific emission potential [20, 35]. In the case of sesquiterpenoids, the term semivolatile has been used to describe their less volatile nature and stickiness to surfaces [36, 37]. For such compounds, temperature influences their re-release from surfaces [37]. Despite the role of temperature in volatile emissions, little is known regarding how temperature influences the actual information-carrying capacity of plant volatiles; for example, could associational resistance as mediated by volatiles be substantially improved or hampered by temperature changes?

*Brassica* plants host a plethora of economically damaging herbivores [12, 38]. Thus, we chose *Brassica oleracea* var. *italica* (broccoli, *B. oleracea* from here on)–*Plutella xylostella*–*Cotesia vestalis* (Haliday) as the agricultural tritrophic model system to be studied along with the well-defended perennial evergreen shrub *Rhododendron tomentosum* Harmaja (previously *Ledum palustre* L.). *Rhododendron tomentosum* is native to northern latitudes and emits specific semivolatiles [palustrol (C_15_H_26_O), ledol (C_15_H_26_O) and ledene (C_15_H_24_)] that are capable of being adsorbed to neighbouring foliage [8]; thus, this species represents a strong semivolatile emitter that is abundant in northern environments, making it interesting to test for its potential for providing associational resistance for use in biocontrol. The diamond-back moth *P. xylostella* L. (Lepidoptera: Yponomeutidae) is a major agricultural pest of *Brassica* with a global distribution [38]. The moth mass migrates by wind currents to northern regions and is attacked by numerous predators and parasitoids, of which the larval parasitoids, e.g., *Cotesia* sp. and *Diadegma* sp. parasitoid wasps, are the most effective for limiting moth population growth [39]. *Cotesia vestalis* (Haliday) (Hymenoptera: Braconidae), a specialist endoparasitoid parasitizing all of the larval instars of *P. xylostella*, uses plant-emitted volatiles in host searching [40].

**Figure 1:**
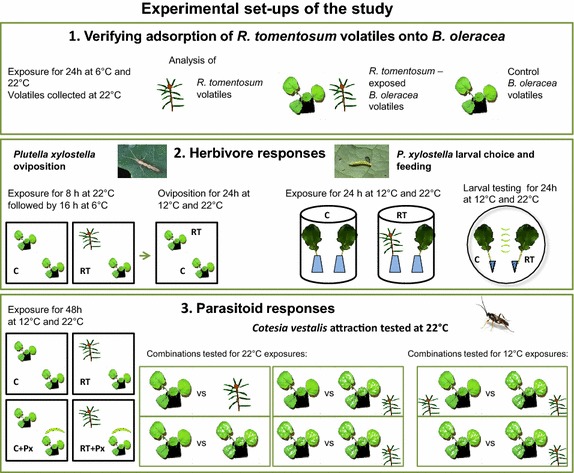
Experimental set-ups of the study. The study consisted of three parts testing plant, herbivore and parasitoid responses towards *R. tomentosum* (RT) exposure. Temperatures used in each part varied according to the response to be tested, and were representative of boreal environment variation in early summer night-time and day-time temperatures. In Part 1 (volatile analysis), a 6°C night-time minimum temperature versus day-time 22°C was used in order to reveal whether a temperature-dependent adsorption–desorption process of RT semivolatiles occurs on *B. oleracea*. Herbivore oviposition in Part 2 was compared at average day-time (22°C) versus average night-time (12°C) temperatures, with equal initial RT exposure (day-time 22°C followed by minimum night-time temperature of 6°C). Larval choice and feeding was tested at stabile 12 and 22°C temperatures to allow comparison for a 24 h period of feeding at night-time versus day-time temperatures along with temperature-representative RT exposures. In Part 3, the day-active parasitoids were tested at 22°C, with the prior RT exposure conducted at average day-time (22°C) and night-time (12°C) temperatures. Px = *P. xylostella*. Photos of *P. xylostella* and *C. vestalis*: Jarmo Holopainen.

Although we were interested in advancing knowledge on the ecological mechanism and temperature sensitivity of associational resistance, our primary aim was to assess the potential of using *R. tomentosum* for biocontrol in this study. The specific objectives of our study were to assess (1) whether associational resistance towards *P. xylostella* is conferred by exposure to *R. tomentosum* in *B. oleracea*; (2) whether *R. tomentosum*-mediated associational resistance is effective both at night-time and day-time temperatures (12 and 22°C upon testing); (3) whether exposure to *R. tomentosum* interferes with indirect defence, i.e., the attraction of the *C. vestalis* parasitoid by host-induced volatiles, of *B. oleracea*; and (4) whether the impact of *R. tomentosum* exposure on indirect defence is affected by temperature (12 versus 22°C) upon exposure. Experimental set-ups of the study are shown in Figure 1 (see “Methods” for details).

## Results

### Verifying the adsorption of *R. tomentosum* volatiles onto neighbouring *B. oleracea*

**Figure 2:**
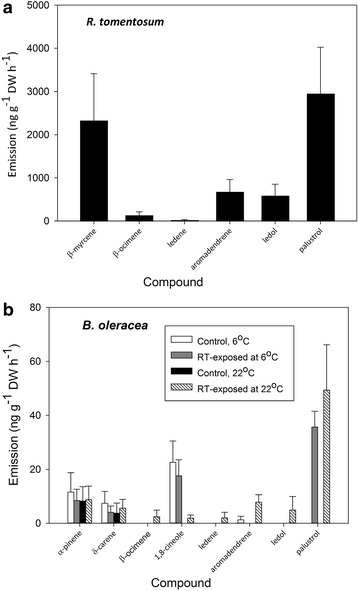
Volatile emission from *Rhododendron tomentosum* (**a**) and control and *R. tomentosum*-exposed *Brassica oleracea* (**b**). The mean ± SEM emission of individual volatile compounds detected are shown. *Brassica oleracea* plants were exposed to *R. tomentosum* for 24 h at 6 or 22°C, and volatile emissions were collected post-exposure at 22°C. *n* = 3 in (**a**) and n = 4 in (**b**) for both exposure temperatures.

We first verified that *R. tomentosum* (RT from here on) exposure resulted in semivolatiles being adsorbed and re-released from *B. oleracea* foliage, as previously reported for birch foliage [8]. RT branch emissions were dominated by the sesquiterpene alcohol palustrol, which comprised 43% of the total volatile emissions (Figure 2a). The other main compounds that were detected were β-myrcene, aromadendrene and ledol (with 34, 10 and 9% of the total emission, respectively). Palustrol was detected from emission profile of RT-exposed *B. oleracea* at both exposure temperatures: 6 and 22°C (Figure 2b). RT-originating ledol and ledene were emitted only from RT-exposed *B. oleracea* plants that were exposed at 22°C. No RT-specific compounds were emitted from the corresponding control *B. oleracea* plants (Figure 2b). There were no statistically significant differences between control and RT-exposed *B. oleracea* in the emissions of volatiles detected from both treatments, i.e., α-pinene, δ-carene and 1,8-cineole (Figure 2b, *P* > 0.05).

### Responses of *P. xylostella* to *R. tomentosum*-exposed *B. oleracea*

**Figure 3:**
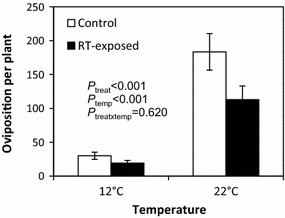
Oviposition by *Plutella xylostella* on control and *R. tomentosum*-exposed *Brassica oleracea* at 12 and 22°C. The values represent the mean number of *P. xylostella* eggs per plant ± SEM based on three independent experiments (*n* = 4 in each experiment). *P* values for the main effects of treatment and temperature and their interaction are shown.

Oviposition by *P. xylostella* was lower on RT-exposed than control *B. oleracea* plants (Figure 3, F_1,36_ = 13.5, *P* < 0.001). At 22°C, there were 38% and, at 12°C, 36% less oviposition on RT-exposed plants. A higher number of eggs were laid when the testing temperature during oviposition was 22°C compared to 12°C (Figure 3, F_1,36_ = 131.6, *P* < 0.001).

**Figure 4:**
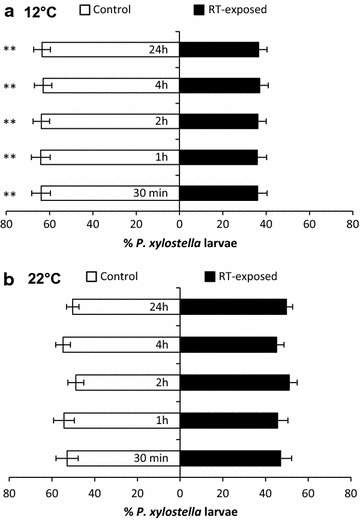
*Plutella xylostella* larval choice towards control versus *R. tomentosum*-exposed *Brassica oleracea* at 12 and 22°C. The percentage distribution (mean ± SEM) of larvae in choice tests performed at 12°C (**a**) and 22°C (**b**) is shown. Each replicate includes the choices (positioning on either leaf) of five individual larvae. *n* = 20–21 in both of the independent replicate experiments for which the results are summarized here. Statistically significant differences in larval choice are indicated by *asterisks* (**P < 0.01).

*Plutella xylostella* larvae preferred to feed on the control rather than the RT-exposed *B. oleracea* leaves at 12°C (Figure 4a, *P* < 0.05). This preference was maintained throughout all of the observation time points from 30 min to 24 h after release. When the exposure and testing took place at 22°C, no significant difference in the choice of the larvae between the control and the RT-exposed *B. oleracea* leaves was detected at any of the observation time points (Figure 4b, *P* > 0.05).

**Figure 5:**
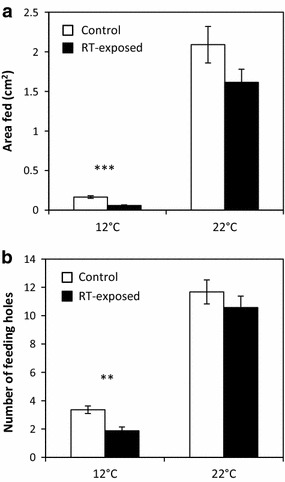
Leaf area fed and feeding holes on control and *R. tomentosum*-exposed *Brassica oleracea.* The amount of leaf area fed at 12°C and at 22°C (**a**) and the number of feeding holes made at 12°C and at 22°C (**b**) (mean ± SEM) by five *Plutella xylostella* larvae over 24 h on control and *R. tomentosum* (RT)-exposed *B. oleracea* leaves are shown. *n* = 20–21 in both of the independent replicate experiments for which the results are summarized here. Statistically significant differences between treatments are indicated by *asterisks* (**P < 0.01, ***P < 0.001).

The leaf area that was consumed by *P. xylostella* larvae was higher in the control than in the RT-exposed *B. oleracea* leaves at 12°C (Figure 5a, *t* = 5.75, df = 41, *P* < 0.001). There was no difference in the amount of leaf damage between the treatments at 22°C (*t* = 1.61, df = 38, *P* = 0.116). The number of feeding holes on RT-exposed *B. oleracea* was smaller than that on control *B. oleracea* leaves at 12°C (Figure 5b, *t* = −3.26, df = 41, *P* = 0.002), whereas no difference between treatments was found when the test took place at 22°C (*t* = −0.95, df = 39, *P* = 0.347).

### Host location by *C. vestalis*: role of *R. tomentosum* exposure

**Figure 6:**
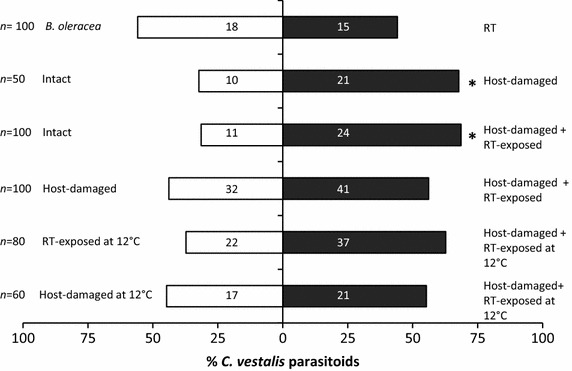
Orientation of parasitoid *Cotesia vestalis* in Y-tube olfactometer assays. The selection of the individually tested parasitoids (% of parasitoids making a selection) towards either of the odour sources in a two-arm olfactometer is shown. Intact, host-damaged and *R. tomentosum* (RT)-exposed refer to treatments of *B. oleracea* plants. Host-damaged plants experienced 48 h of *Plutella xylostella* L. larval feeding and RT-exposed plants neighboured a potted *R. tomentosum* plant for 48 h before testing at the temperature shown. The total number of parasitoids tested (*n*) and number of individuals making a selection towards the odour sources are also reported. Statistically significant differences in the selections are indicated by *asterisks* (*P < 0.05).

*Cotesia vestalis* females showed no preference for intact *B. oleracea* over RT volatiles (Figure 6, χ^2^ = 0.27, *P* = 0.602), and only 33% of the parasitoids made a choice in this behavioural test comparison. The functioning of the *B. oleracea*–*P. xylostella*–*C. vestalis* tritrophic interaction was verified by the greater attraction of *C. vestalis* females towards host-damaged rather than intact *B. oleracea* volatiles (χ^2^ = 3.90, *P* = 0.048). The parasitoids also preferred host-damaged, RT-exposed *B. oleracea* over intact plants when the exposure took place at day-time temperature (22°C) (χ^2^ = 4.83, *P* = 0.028). No preference by *C. vestalis* was observed between host-damaged and host-damaged RT-exposed *B. oleracea* at this temperature (χ^2^ = 1.11, *P* = 0.292). The parasitoid choosing rates were greater than 60% in all three assays with host-damaged *B. oleracea* as an odour source.

When using plants that were exposed to RT at 12°C, *C. vestalis* preferred host-damaged RT-exposed *B. oleracea* plants to intact RT-exposed plants with a marginal statistical significance (Figure 6, χ^2^ = 3.81, *P* = 0.067). The parasitoid had no preference for host-damaged over host-damaged RT-exposed *B. oleracea* (χ^2^ = 0.24, *P* = 0.743).

## Discussion

Our results revealed that RT exposure reduced the oviposition and larval feeding of a key pest herbivore of cruciferous plants: the diamondback moth *P. xylostella* on *B. oleracea.* A colder temperature (12°C) upon exposure was integral to the increase in larval resistance. In addition, indirect defence, evaluated here as the attraction of the specialist parasitoid *C. vestalis* by host-induced volatiles, remained persistent despite RT exposure, although indications for a reduced response were found when the exposure took place at a colder temperature (12°C).

### Associational resistance via *R. tomentosum*: mechanisms of action and potential for biocontrol

The provision of associational resistance by RT volatiles, as previously suggested for a natural neighbour (*Betula* sp.) [8], was confirmed in this study on a *Brassica* crop species and towards a damaging global pest: the diamondback moth. In extracts of RT, with ledene, ledol and palustrol forming the majority of the composition, repellence has been reported towards various arthropods [41, 42]. Our finding that exposure via air alone was sufficient to render increased resistance towards both adult and larval stages of *P. xylostella*, with no need for the physical application of the compounds, suggests that RT could be a pest-suppressive intercrop for *Brassica*.

Our study is not able to determine whether the increased resistance acts as an olfactory, as an contact cue or both. Furthermore, the mode of action for RT exposure leading to associational resistance on *B. oleracea* also remains an open question: the observed impacts could stem from RT volatiles that are adsorbed onto and re-released from *B. oleracea* leaves or from *B. oleracea* volatiles induced by RT exposure. Because *B. oleracea* intrinsic volatile emission was low and there were no indications of other compounds being altered in the profile after RT exposure than the RT-specific semivolatiles, which can be adsorbed onto foliage [8], the latter hypothesis is not supported. Therefore, RT volatiles are likely candidates for passively providing increased resistance. These persistent compounds, which are emitted in large amounts by RT, could disguise host location, oviposition or feeding stimulation cues, such as glucosinolate break-down products for the specialist pest *P. xylostella* [39]. Alternatively, these compounds might cause direct repellence [41, 42]. Thus, follow-up laboratory work with synthetic palustrol, ledol and ledene is needed to reveal the acting mechanism for RT-exposure-mediated associational resistance.

The potential importance of RT semivolatiles is further supported by the observed temperature dependence of the resistance towards larval feeding: at 12°C, the RT specific semivolatiles are stuck on surfaces. In [37], an effort to establish artificial broccoli leaves, i.e., a wax layer on a microscope slide, for detecting adsorption of induced volatiles, showed how induced sesquiterpenes in particular were adsorbed onto the wax and then re-released upon increasing temperature. As epicuticular wax is the surface encountering RT volatiles under aerial exposure, their mode of action could involve an interaction with wax chemicals. *Plutella xylostella* prefers to oviposit on glossy versus waxy leaves, and high epicuticular wax bloom makes cabbage more resistant for the pest [43]. The chemical composition of the wax varies between the types, i.e., the proportion of alkanes and ketones is lower, while that of fatty acids and triterpenoids is larger on glossy wax type leaves [44]. In addition, also *P. xylostella* larvae are impacted by the wax properties: they spend more time searching and less time feeding on glossy cabbages [44]. Thus, one hypothesis for the mechanism for the partly temperature-dependent RT-mediated resistance in our study is the chemical compositional changes that are induced by RT volatiles in *B. oleracea* leaf wax.

In biocontrol, companion crops with reported impacts on agriculturally important herbivores have often included aromatic-volatile-emitting herbs [45, 46], indicating that the potential semivolatile characteristics that their main volatiles possess might be more generally important for the effectiveness of associational resistance. However, the effectiveness of herb intercropping impacting herbivore abundance varies [12, 46]. Additional variability complicating understanding of the role of volatiles for associational resistance is created by the differential behavioural patterns that are likely observed for generalist versus specialist herbivores [12]. In addition, there is ample variation by species in utilising volatiles in host location [6] in comparison to other cues, such as phenotypic differences acting as physical barriers or visual cues [17, 47]. Our finding that temperature impacts the effectiveness of associational resistance emphasizes the need to include a more detailed analysis of changes in volatile emissions under natural temperature variations considering their properties affecting their volatility or adsorption onto foliage [36, 37]. The volatile profile of a plant species is a genetically regulated property that is important to be assessed when developing agricultural applications based on associational resistance [15, 16].

### Persistent indirect defence: does high olfactory specificity at upper trophic levels protect against interference?

Indirect defence, assessed here for the attraction of a specialist parasitoid *C. vestalis* to host-damaged *B. oleracea* volatiles, was not interfered with by RT exposure at 22°C. The attraction was slightly reduced following the 12°C exposure, indicating that RT exposure might become more meaningful at morning and late evening hours when the temperature decreases. The greater tendency for the studied herbivore to be influenced by RT exposure rather than its natural enemy is supported by a field study in which there was no difference in parasitism, but densities of larval and pupal stages of *P. xylostella* were reduced in the mixed cropping of cabbage and coriander [47]. The ability of a non-host scent to act towards both a herbivore and its natural enemy, has been reported for a Yeheb (*Cordeauxia edulis*) plant extract [48] which at the same time attracted *C. vestalis* and repelled *P. xylostella*. We found no such attracting effect from RT towards *C. vestalis* that could have led to synergy in *P. xylostella* biocontrol.

To our knowledge, the passive adsorption of heterospecific species emitted volatiles, and the resulting associational resistance has not been considered in the context of disturbing indirect defence earlier. However, the abundance and foraging efficiency of parasitoids have been studied in complex versus less complex environments [49–51], with chemical complexity hypothesized to be one influencer of arthropod abundance [50]. The parasitoid *Diadegma semiclausum* entered a *Sinapis*–*Brassica* intercrop faster than a *Hordeum*–*Brassica* intercrop, but took more time to find host there [49], indicating that species composition is meaningful, even though the co-crop volatiles were not analysed, permitting a comparison of volatile-mediated and other cues. In [51], as the habitat complexity increased, stronger host cues for *C. glomerata* host finding were concluded to be required. In addition, in [52], *C. glomerata*, with a wider host range compared to that of the more specialised *Cotesia rubecula*, was less effective in host finding in mixed cultures of *B. oleracea* and potato. In a laboratory study with volatile interference introduced by isoprene, another parasitoid-specific response was found: isoprene interfered with the host location in *D. semiclausum* but not in *C. rubecula* [22]. The mixing of non-host herbivore-induced cabbage volatiles and host-induced volatiles of bean did not interfere with the attraction of specialist predatory mites to prey [53]. In [54], non-host volatiles were verified to be sensed by a predaceous beetle, suggesting the ability of non-host volatiles in particular to hamper tritrophic interactions. It could be that more specialised parasitoids, such as *C. vestalis* in our study, are less sensitive to disturbance than herbivore natural enemies with a wider host range and thus a higher tendency for adaptation. Thus, it will be interesting to test in the future whether the specialization of the tritrophic interaction plays a key role in the sensitivity to neighbour plant semivolatile interference.

### Temperature as a mediator of associational resistance

Temperature varies greatly in nature and in agroecosystems, diurnally, seasonally and regionally, impacting plant phenology and insect activity and, thus, their interactions [55]. Night-time temperatures often fall close to 10°C in boreal regions (the natural habitats of RT) in the summer. Thus, nocturnal and day-active herbivores might be variably affected by exposure to RT semivolatiles and their presence in air versus foliage. *Plutella xylostella* moths are night-active, and their oviposition peaks at dusk, but light during night (as typical for boreal summer) does not inhibit oviposition [39]. Increasing temperature hastens the duration of the life cycle of *P. xylostella* as well as its oviposition and larval feeding activity [39]. The reported equal reduction in oviposition on RT-exposed plants under both studied temperatures suggests a diurnally persistent increase in resistance towards *P. xylostella*. Oviposition is agronomically important because it determines the initial acceptance of the host plant. *Plutella xylostella* does not oviposit on non-host plants as it relies on a variety of chemical and physical host recognition cues [56]; RT exposure seems to interfere with one or several of these.

Our result on resistance towards *P. xylostella* larval feeding emerging at a lower temperature is less beneficial for the plant as *P. xylostella* feeding increases by temperature [39]. However, the finding is interesting with regards to hypothesizing a regulating role for temperature in semivolatile-mediated associational resistance. The temperature dependence of the volatiles acting might offer one hypothetical explanation for why aromatic herbs that are rich in sesquiterpenes have often shown success, but also failures in providing resistance in mixed cropping [12, 14–16]. To reveal whether this is the case, the leaf-air interface dynamics of the specific volatiles acting should be studied in detail under temperature regimes.

Another consideration in the long-term effectiveness of associational resistance as conferred by the neighbour’s volatiles is that the non-static nature of the signal, mediated by temperature, might also help slow both the adaptation of herbivores to the scent [57] and the evolution of breaking host plant resistance by herbivores. Temperature control renders the signal most effective at certain time periods only, partly resembling the mode of function of inducible defences [23]. Both mechanisms might be ecologically and even evolutionarily supported by the dynamic nature of signal appearance in addition to saving resources from the receiver.

The overall effect of temperature variations on the plant-herbivore dynamics in a multitrophic system depends on the comparative responses of herbivores and their natural enemies. The temperature sensitivity of different trophic-level insects varies [58]; parasitoids and predators are generally assumed to be more responsive than their prey, as they rely more on mobility in prey searching and are physiologically more responsive to temperature. Upon temperature changes, herbivores, on the other hand, face bottom-up changes in plant host quality, show direct physiological and reproductional responses [55], plus are confronted by altered top-down control from natural enemies [59]. The parasitoid studied here, *C. vestalis*, oviposits upon photophase with optimal temperature for parasitism at 20–35°C [60]. Thus, our finding that the host location by *C. vestalis* was not interfered with by RT exposure at 22°C suggests that using RT for *P. xylostella* biocontrol should not compromise the ability of *C. vestalis* to locate host on *B. oleracea*. The slightly reduced attraction towards plants that were exposed to RT at 12°C suggests, however, that exposure to RT might affect the interaction at low temperatures such as during early morning.

## Conclusions

The ability of RT exposure, earlier discovered to render associational resistance in nature, to influence *Brassica* herbivore oviposition and larval feeding encourages further testing of its potential for biocontrol. The generality of our finding on the regulatory role of temperature on associational resistance should be tested in the future on natural and agricultural plant systems varying in plant volatile composition (acknowledging the differential role of semivolatile versus more volatile compounds). Additionally, the level of specialisation and the use of the visual, olfactory and chemoreceptor cues of targeted herbivores should be studied together to distinguish the role of volatile-based and other neighbouring potentiated defences. Finally, our study emphasizes that for developing biocontrol applications based on associational resistance, more attention needs to be paid to the compatibility of plant defences with herbivore diurnal behaviour and times of activity as well as that of their natural enemies.

## Methods

### Plants, herbivores and natural enemies

*Brassica oleracea* var. *italica* (broccoli) cv. Lucky seedlings were grown from seed in 0.66 l pots (4 plants in each, mixture 2:1:1 of fertilized compost, NPK 100:30:200 mg l^−1^: B2 peat NPK 110:40:220 mg l^−1^: sand) in a greenhouse (temperature 20/16°C day/night) and used at approximately 3 weeks of age for experiments. *R. tomentosum* (RT) branches that were collected from a natural habitat in Suonenjoki, Finland (62°38′42.683″N, 27°3′55.383″E) and stored at 6°C prior to use if necessary, were used in the exposure treatments. Potted greenhouse-grown RT plants (seed origin: natural habitat in Neulaniemi, Kuopio, grown in 3:1 peat:sand) of circa 20 cm in height were used in the Y-tube olfactometer tests. The *P. xylostella* larvae and adults and *C. vestalis* parasitoids that were used in the experiments originated from laboratory populations maintained on *B. oleracea* var. *italica* (20–25°C temperature and 16L:8D photoperiod) at the University of Eastern Finland.

### Volatile collection and analysis

For exposure, four *B. oleracea* pots (with four 21-days-old plants/pot) were enclosed in insect cages (33 × 33 × 60 cm, with two sides covered with a fabric mesh) with 15 cut hibernating RT branches (collected right before the experiments from the natural habitat) that had their stems in water-filled 250-ml decanter bottles. Control cages had an equal set-up but hosted no RT branches. The cages were placed at 6°C (cold room) and 22°C (a laboratory fume hood) under low light (approximately 150 µmol m^−2^ s^−1^). The colder night temperature was used to mimic minimum boreal early summer temperatures. After 24 h, the plants were removed from exposure, and the volatiles were collected at 22°C using a dynamic bag enclosure method. The RT branch emissions were collected from three branches at 22°C to determine their characteristic emission profile.

For volatile collection, the entire shoot of a five-leaf stage broccoli plant or RT branch was enclosed inside pre-cleaned (heating for 1 h at 120°C) multipurpose cooking bags (polyethylene terephthalate (PET), 25 × 55 cm in size, Look, Terinex Ltd, UK), and the bag opening was tied around the base of the stem with a piece of thin garden wire. After the enclosure of the plant, air that had been filtered through charcoal and a MnO_2_ scrubber were pumped into bags through an opening in one of the top corners of the PET bags at a flow rate of 600 ml min^−1^ for 10 min to flush out residual contaminants. The sampling line was inserted into another corner of the bag and tied with a wire. After fixing the lines, the inflow rate was reduced to 300 ml min^−1^, and the volatile emissions were sampled in stainless steel tubes that were filled with 150 mg of Tenax TA adsorbent (Supelco, Bellefonte, PA USA) for 30 min at the rate of about 200 ml min^−1^ with a vacuum pump (Thomas 5002 12V DC). An equal light level (approximately 300 µmol m^−1^ s^−1^) was provided by placing two lamps (Lival Shuttle Plus, Lival Oy, Sipoo, Finland with Osram Delux F, 24W fluorescent lamps, Osram AG, Munich, Germany) on both sides of each plant. Sample tubes were sealed with Teflon-coated brass caps immediately after collection and stored in the refrigerator until analysis. The plant biomass was oven-dried (60°C) to determine the dry weights of the plants.

The plant volatile samples were analysed by a gas chromatograph-mass spectrometer (GC type 6890, MSD 5973: Hewlett Packard; Wilmington, DE, USA) as described in [61]. The trapped compounds were desorbed with a thermal desorption unit (Perkin-Elmer ATD400 Automatic Thermal Desorption system) at 250°C for 10 min, cryofocused at −30°C and injected onto a HP-5 capillary column (50 m × 0.2 mm i.d. × 0.5 μm film thickness, Hewlett-Packard) with helium as a carrier gas. The oven temperature programme was held at 40°C for 1 min and then increased to 210°C at a rate of 5°C min^−1^ and finally to 250°C at a rate of 20°C min^−1^. The compounds were identified and quantified by comparing the spectra of external standards for available compounds and the Wiley library (John Wiley & Sons, Ltd, Chichester, UK). The emission rates were calculated as emission per dry weight (DW) per hour.

### Oviposition by *P. xylostella* moths

RT exposure was identical in the set-up as used for the plants for volatile analysis. Here, the exposure was maintained (as well as the corresponding control plants with no RT branches inside cages) in fume hoods at 22°C for the previous day (8 h) followed by 16 h at 6°C (cold room, representing typical early summer night-time minimum temperature) until the start of the oviposition experiment. All of the plants were equally exposed to assure equal initial amounts of potentially adsorbed semivolatiles by exposure, revealing solely day-time versus night-time temperature differences for oviposition.

The oviposition in control and RT-exposed *B. oleracea* was experimented in 2.6 m^3^ temperature-controlled growth chambers (described in [62]) at temperatures of 12 and 22°C (mimicking average boreal night-time and day-time temperatures, respectively) and a 22L:2D photoperiod with approximately 250 µmol m^−2^ s^−1^ light level. One control and one RT-exposed *B. oleracea* pot were enclosed in each of the four replicate insect cages under both of the temperature treatments. Thirty *P. xylostella* moths were randomly selected by the expectation of the 50:50 female:male ratio (population well stabilised to assure that both sexes were present and allowing mating as in nature) [48] and released into each cage. After 24 h, the *B. oleracea* plants were removed from cages and stored at 6°C until egg counting. Three independent replicate experiments were carried out.

### Host choice and feeding by *P. xylostella* larvae

For herbivore choice tests, the exposure of *B. oleracea* leaves to RT volatiles was conducted in 1.5-l glass containers holding two 30-ml glass vials approximately 3 cm apart, as in [8]. Ten to eleven independent systems were built per replicate experiment (two independent replicate experiments were conducted for both temperatures), each having two *B. oleracea* leaves in one vial and either an RT branch (RT-exposure treatment) or a *B. oleracea* leaf (control) in the other vial. The exposures and the following choice tests took place for 24 h for each part in a laboratory fume hood (22°C) and a cold room (12°C), mimicking average boreal early summer day-time and night-time temperatures, respectively.

For choice tests, the petioles of *B. oleracea* leaves (one each from control and exposed system set-ups) were inserted with forceps into water-filled 1.5-ml Eppendorf tubes through a hole in the cap and placed into 13-cm-diameter glass Petri dishes that were lined with filter paper. An equal light level (approximately 300 µmol m^−1^ s^−1^) was provided by placing four fluorescent lamps above the dishes. The position of the control and exposed leaves was alternated in order to remove any remaining inequality in light conditions. Five 2nd instar *P. xylostella* larvae were released in the middle of each dish, and the location of the herbivores on each leaf was recorded at 30 min, 1, 2, 4 and 24 h. After 24 h, the larvae were removed, and each leaf was photographed to determine the number of feeding holes and the leaf area that was consumed by the larvae in pixels, which was converted to square centimetres using Adobe Photoshop Elements 2.0 (Adobe Systems Incorporated, Wilmington, DE, USA).

### Host location by *C. vestalis* females

The potential interference on the olfactory orientation of *C. vestalis* towards *P. xylostella*-induced *B. oleracea* volatiles by RT was tested using 48 h exposure treatments at 22 and 12°C (average boreal early season day-time and night-time temperatures, respectively). For RT exposure treatments, *B. oleracea* plants in pots (4 per pot, 21 days old) were placed within a 10-cm distance of a RT plant inside plastic insect cages (as previously described) in a fume hood at 22°C or a cold room at 12°C. For host herbivore damage treatments, a total of three 4th instar and three 2nd instar *P. xylostella* larvae were placed in each pot of *B. oleracea*. An equal number of same-aged plants were used for the control treatment. The larvae were allowed to feed for 48 h, after which they were removed immediately prior to behavioural tests.

The plants (one pot per enclosure) with the soil and pot covered with aluminium foil were placed inside 22-l glass vessels immediately after ending the treatments and used as odour sources to assess the parasitoid orientation in a Y-tube olfactometer. The plants that were exposed at 12°C were put inside the vessels at 12°C and immediately before testing transferred to the laboratory where olfactometry tests were conducted with *C. vestalis* at 22°C.

Four comparisons were tested using plant treatments at 22°C: (1) intact *B. oleracea* versus RT (to determine whether *C. vestalis* is intrinsically affected by RT volatiles); (2) intact *B. oleracea* versus host-damaged *B. oleracea* (to verify the functioning of the system for testing orientation of the parasitoid to host damage); (3) intact *B. oleracea* versus host-damaged RT-exposed *B. oleracea* (to determine whether RT exposure disturbs the olfaction of induced volatiles); and (4) host-damaged *B. oleracea* versus host-damaged RT-exposed *B. oleracea* (to determine whether *C. vestalis* is able to discriminate between these). With the plants from 12°C treatments, two comparisons were tested: (5) RT-exposed *B. oleracea* versus host-damaged RT-exposed *B. oleracea* (to determine whether RT exposure at 12°C disrupts host finding) and (6) host-damaged *B. oleracea* versus host-damaged RT-exposed *B. oleracea* (testing the ability to discriminate between these after exposure at 12°C).

Two-to-five days old mated female parasitoids, which were provided with a minimum 2-h-long learning period (improving the responsiveness of the parasitoid, [40]) with a *P. xylostella*-infested *B. oleracea* plant prior to testing, were used in the Y-tube olfactometer assays. For each test, female *C. vestalis* parasitoids were introduced individually downwind at the opening of the Y-tube, with arms connected to the two 22-l glass vessel odour sources (see [40] for details). A choice was recorded when the parasitoid had reached the last third of either arm and stayed there for more than 3 s. The maximum time for recording a choice was 5 min. Ten individuals were tested with the same odour sources, and each parasitoid was tested only once. A total of 50–100 parasitoids were tested for each comparison.

### Statistical analyses

All data were checked for normality and equality of variances before statistical analysis and log-transformed to meet the requirements when needed. Differences in concentrations of individual volatile compounds (detected in both of the treatments) were analysed with independent samples *t* tests, separately for the two exposure temperatures. *Plutella xylostella* oviposition data were analysed for the main effects of treatment, temperature and their interaction, including the experiment as a random effect, using a mixed model for log-transformed values. Paired samples *t* tests were used to test *P. xylostella* larval choice, leaf area removed and the number of feeding holes separately for the two temperatures. Both of the replicate experiments were analysed together. Chi square tests (comparison to the binomial distribution) were used to analyse the Y-tube olfactometer choice test data. All tests were performed with SPSS 14.0 for Windows (SPSS Inc., Chicago, IL, USA).

## Abbreviation

RT: *Rhododendron tomentosum* (Harmaja)

## References

[1] Tahvanainen JO, Root RB (1972). The influence of vegetational diversity on the population ecology of a specialized herbivore, *Phyllotreta cruciferae* (Coleoptera: Chrysomelidae). Oecologia.

[2] Atsatt PR, O’Dowd DJ (1976). Plant defense guilds. Science.

[3] Root RB (1973). Organization of a plant-arthropod association in simple and diverse habitats: The fauna of collards (*Brassica oleracea*). Ecol Monogr.

[4] Altieri MA, Whitcomb WH (1979). Potential use of weeds in the manipulation of beneficial insects. HortSci.

[5] Dicke M (2000). Chemical ecology of host-plant selection by herbivorous arthropods: a multitrophic perspective. Biochem Syst Ecol.

[6] Bruce TJA, Wadhams LJ, Woodcock CM (2005). Insect host location: a volatile situation. Trends Plant Sci.

[7] Karban R (2011). The ecology and evolution of induced resistance against herbivores. Funct Ecol.

[8] Himanen SJ, Blande JD, Klemola T, Pulkkinen J, Heijari J, Holopainen JK (2010). Birch (*Betula* sp.) leaves adsorb and re-release volatiles specific to neighbouring plants—a mechanism for associational herbivore resistance?. New Phytol.

[9] Barbosa P, Hines J, Kaplan I, Martinson H, Szczepaniec A, Szendrei Z (2009). Associational resistance and associational susceptibility: having right or wrong neighbors. Annu Rev Ecol Evol Syst.

[10] Jactel H, Birgersson G, Andersson S, Schlyter F (2011). Non-host volatiles mediate associational resistance to the pine processionary moth. Oecologia.

[11] Zakir A, Sadek MM, Bengtsson M, Hansson BS, Witzgall P, Anderson P (2013). Herbivore-induced plant volatiles provide associational resistance against an ovipositing herbivore. J Ecol.

[12] Hooks CRR, Johnson MW (2003). Impact of agricultural diversification on the insect community of cruciferous crops. Crop Prot..

[13] Newton AC, Begg GS, Swanston JS (2009). Deployment of diversity for enhanced crop function. Ann Appl Biol..

[14] Andow DA (1991). Vegetational diversity and arthropod population response. Annu Rev Entomol.

[15] Song BZ, Wu HY, Kong Y, Zhang J, Du YL, Hu JH (2010). Effects of intercropping with aromatic plants on the diversity and structure of an arthropod community in a pear orchard. Biocontrol.

[16] Mauchline AL, Cook SM, Powell W, Osborne JL (2013). Effects of non-host plant odour on *Meligethes aeneus* during immigration to oilseed rape. Entomol Exp Appl.

[17] Finch S, Collier RH (2012). The influence of host and non-host companion plants on the behaviour of pest insects in field crops. Entomol Exp Appl.

[18] Yuan JS, Himanen SJ, Holopainen JK, Chen F, Stewart CN (2009). Smelling global climate change: mitigation of function for plant volatile organic compounds. Trends Ecol Evol.

[19] Blande JD, Holopainen JK, Li T (2010). Air pollution impedes plant-to-plant communication by volatiles. Ecol Lett.

[20] Holopainen JK, Gershenzon J (2010). Multiple stress factors and the emission of plant VOCs. Trends Plant Sci.

[21] Heil M (2014). Herbivore-induced plant volatiles: targets, perception and unanswered questions. New Phytol.

[22] Loivamäki M, Mumm R, Dicke M, Schnitzler JP (2008). Isoprene interferes with the attraction of bodyguards by herbaceous plants. Proc Natl Acad Sci USA.

[23] Dicke M, Baldwin IT (2010). The evolutionary context for herbivore-induced plant volatiles: beyond the ‘cry for help’. Trends Plant Sci.

[24] Shelton AM, Badenes-Perez FR (2006). Concepts and applications of trap cropping in pest management. Annu Rev Entomol.

[25] Cook SM, Khan ZR, Pickett JA (2007). The use of push-pull strategies in integrated pest management. Annu Rev Entomol.

[26] Kaplan I (2012). Attracting carnivorous arthropods with plant volatiles: the future of biocontrol or playing with fire?. Biol Control.

[27] Cook SM, Jönsson M, Skellern MP, Murray DA, Anderson P, Powell W (2007). Responses of *Phradis* parasitoids to volatiles of lavender, *Lavandula angustifolia*—a possible repellent for their host, *Meligethes aeneus*. BioControl.

[28] Glinwood R, Ninkovic V, Pettersson J (2011). Chemical interaction between undamaged plants—effects on herbivores and natural enemies. Phytochemistry.

[29] Wäschke N, Meiners T, Rostás M, Wajnberg E, Colazza S (2013). Foraging strategies of parasitoids in complex chemical environments. Chemical ecology of insect parasitoids.

[30] Vucetic A, Dahlin I, Petrovic-Obradovic O, Glinwood R, Webster B, Ninkovic V (2014). Volatile interaction between undamaged plants affects tritrophic interactions through changed plant volatile emission. Plant Signal Behav..

[31] Braasch J, Kaplan I (2012). Over what distance are plant volatiles bioactive? Estimating the spatial dimensions of attraction in an arthropod assemblage. Entomol Exp Appl.

[32] Kesselmeier J, Staudt M (1999). Biogenic volatile organic compounds (VOC): an overview on emission, physiology and ecology. J Atmos Chem.

[33] Niinemets Ü, Loreto F, Reichstein M (2004). Physiological and physicochemical controls on foliar volatile organic compound emissions. Trends Plant Sci.

[34] Peñuelas J, Staudt M (2010). BVOCs and global change. Trends Plant Sci.

[35] Harrison SP, Morfopoulos C, Dani KGS, Prentice IC, Arneth A, Atwell BJ (2013). Volatile isoprenoid emissions from plastid to planet. New Phytol.

[36] Helmig D, Bocquet F, Pollmann J, Revermann T (2004). Analytical techniques for sesquiterpene emission rate studies in vegetation enclosure experiments. Atmos Environ.

[37] Li T, Blande JD (2015). Associational susceptibility in broccoli: mediated by plant volatiles, impeded by ozone. Glob Chang Biol.

[38] Furlong MJ, Wright DJ, Dosdall LM (2013). Diamondback moth ecology and management: Problems, progress, and prospects. Annu Rev Entomol.

[39] Talekar NS, Shelton AM (1993). Biology, ecology, and management of the diamondback moth. Annu Rev Entomol.

[40] Himanen SJ, Nerg AM, Nissinen A, Pinto DM, Stewart CN, Poppy GM (2009). Effects of elevated carbon dioxide and ozone on volatile terpenoid emissions and multitrophic communication of transgenic insecticidal oilseed rape (*Brassica napus* L.). New Phytol.

[41] Jaenson T, Palsson K, Borg-Karlson A (2005). Evaluation of extracts and oils of tick-repellent plants from Sweden. Med Vet Entomol.

[42] Egigu MC, Ibrahim MA, Yahya A, Holopainen JK (2011). *Cordeauxia edulis* and *Rhododendron tomentosum* extracts disturb orientation and feeding behavior of *Hylobius abietis* and *Phyllodecta laticollis*. Entomol Exp Appl.

[43] Justus KA, Dosdall LM, Mitchell BK (2000). Oviposition by *Plutella xylostella* (Lepidptera: Plutellidae) and effects of phylloplane waxiness. J Econ Entomol.

[44] Eigenbrode SD, Espelie KE, Shelton AM (1991). Behavior of neonate diamondback moth larvae (*Plutella xylostella* L.) on leaves and on extracted leaf waxes of resistant and susceptible cabbages. J Chem Ecol.

[45] Mauchline AL, Osborne JL, Martin AP, Poppy GM, Powell W (2005). The effects of non-host plant essential oil volatiles on the behaviour of the pollen beetle *Meligethes aeneus*. Entomol Exp Appl.

[46] Tang GB, Song BZ, Zhao LL, Sang XS, Wan HH, Zhang J (2013). Repellent and attractive effects of herbs on insects in pear orchards intercropped with aromatic plants. Agroforest Syst.

[47] Adati T, Susila W, Sumiartha K, Sudiarta P, Toriumi W, Kawazu K (2011). Effects of mixed cropping on population densities and parasitism rates of the diamondback moth, *Plutella xylostella* (Lepidoptera: Plutellidae). Appl Entomol Zool.

[48] Egigu MC, Ibrahim MA, Yahya A, Holopainen JK (2010). Yeheb (*Cordeauxia edulis*) extract deters feeding and oviposition of *Plutella xylostella* and attracts its natural enemy. Biocontrol.

[49] Gols R, Bukovinszky T, Hemerik L, Harvey JA, van Lenteren JC, Vet LEM (2005). Reduced foraging efficiency of a parasitoid under habitat complexity: implications for population stability and species coexistence. J Anim Ecol.

[50] Randlkofer B, Obermaier E, Hilker M, Meiners T (2010). Vegetation complexity—the influence of plant species diversity and plant structures on plant chemical complexity and arthropods. Basic Appl Ecol.

[51] Bezemer TM, Harvey JA, Kamp AFD, Wagenaar R, Gols R, Kostenko O (2010). Behaviour of male and female parasitoids in the field: influence of patch size, host density, and habitat complexity. Ecol Entomol.

[52] Perfecto I, Vet LEM (2003). Effect of a nonhost plant on the location behavior of two parasitoids: the tritrophic system of *Cotesia* spp. (Hymenoptera: Braconidae), *Pieris rapae* (Lepidoptera: Pieridae), and *Brassica oleraceae*. Environ Entomol.

[53] Dicke M, de Boer JG, Höfte M, Rocha-Granados MC (2003). Mixed blends of herbivore-induced plant volatiles and foraging success of carnivorous arthropods. Oikos.

[54] Zhang QH, Schlyter F (2010). Inhibition of predator attraction to kairomones by non-host plant volatiles for herbivores: a bypass trophic signal. PLoS One.

[55] Bale JS, Masters GJ, Hodkinson ID, Awmack C, Bezemer TM, Brown VK (2002). Herbivory in global climate change research: direct effects of rising temperature on insect herbivores. Glob Chang Biol..

[56] Sarfraz M, Dosdall LM, Keddie BA (2006). Diamondback moth-host plant interactions: Implications for pest management. Crop Prot..

[57] Simoes P, Ott SR, Niven JE (2011). Associative olfactory learning in the desert locust, *Schistocerca gregaria*. J Exp Biol.

[58] Voigt W, Perner J, Davis AJ, Eggers T, Schumacher J, Bährmann R (2003). Trophic levels are differentially sensitive to climate. Ecology.

[59] Berggren Å, Björkman C, Bylund H, Ayres MP (2009). The distribution and abundance of animal populations in a climate of uncertainty. Oikos.

[60] Talekar NS, Yang JC (1991). Characteristic of parasitism of diamondback moth by two larval parasites. Entomophaga.

[61] Ibrahim MA, Mäenpää M, Hassinen V, Kontunen-Soppela S, Malec L, Rousi M (2010). Elevation of night-time temperature increases terpenoid emissions from *Betula pendula* and *Populus tremula*. J Exp Bot.

[62] Vuorinen T, Nerg A-M, Ibrahim MA, Reddy GVP, Holopainen JK (2004). Emission of *Plutella xylostella*-induced compounds from cabbages grown at elevated CO_2_ and orientation behavior of the natural enemies. Plant Physiol.

